# Comparing Quick Sequential Organ Failure Assessment Scores to End-tidal Carbon Dioxide as Mortality Predictors in Prehospital Patients with Suspected Sepsis

**DOI:** 10.5811/westjem.2018.1.35607

**Published:** 2018-03-13

**Authors:** Christopher L. Hunter, Salvatore Silvestri, George Ralls, Amanda Stone, Ayanna Walker, Neal Mangalat, Linda Papa

**Affiliations:** * Orlando Regional Medical Center, Department of Emergency Medicine, Orlando, Florida; † University of Central Florida College of Medicine, Department of Emergency Medicine, Orlando, Florida; ‡ St Mary’s Hospital, Department of Emergency Medicine, St. Louis, Missouri

## Abstract

**Introduction:**

Early identification of sepsis significantly improves outcomes, suggesting a role for prehospital screening. An end-tidal carbon dioxide (ETCO_2_) value ≤ 25 mmHg predicts mortality and severe sepsis when used as part of a prehospital screening tool. Recently, the Quick Sequential Organ Failure Assessment (qSOFA) score was also derived as a tool for predicting poor outcomes in potentially septic patients.

**Methods:**

We conducted a retrospective cohort study among patients transported by emergency medical services to compare the use of ETCO_2_ ≤ 25 mmHg with qSOFA score of ≥ 2 as a predictor of mortality or diagnosis of severe sepsis in prehospital patients with suspected sepsis.

**Results:**

By comparison of receiver operator characteristic curves, ETCO_2_ had a higher discriminatory power to predict mortality, sepsis, and severe sepsis than qSOFA.

**Conclusion:**

Both non-invasive measures were easily obtainable by prehospital personnel, with ETCO_2_ performing slightly better as an outcome predictor.

## INTRODUCTION

Early identification and treatment of sepsis, including timely administration of intravenous fluids and antibiotics, has shown to significantly improve outcomes.^1^–^3^ Many septic patients receive their initial care from prehospital personnel, providing an opportunity for early detection.^4^,^5^ Hallmarks of severe sepsis include hypoperfusion, lactic acidosis, and organ failure. Exhaled end-tidal carbon dioxide (ETCO_2_) has a negative correlation with serum lactate levels and a similar predictive value for poor outcomes in suspected sepsis.^6^ In fact, prehospital ETCO_2_ values of ≤ 25 mmHg may predict mortality and severe sepsis as part of a screening tool for potentially septic patients.^7^,^8^ Recently, the Quick Sequential Organ Failure Assessment (qSOFA) score was derived as a tool for predicting poor outcomes, defined as mortality or admission to the intensive care unit (ICU) for ≥ 3 days, in patients with suspected sepsis.^9^

The qSOFA score is calculated by using altered mental status (defined by Glasgow Coma Scale [GCS] < 15), systolic blood pressure (SBP) ≤ 100 mm Hg, and respiratory rate (RR) ≥ 22 breaths per minute. Retrospective analysis suggests a qSOFA score of two or greater is associated with a high risk of poor outcomes. This score can be quickly calculated without the need for laboratory values, so it may have utility in the prehospital environment. This study aims to compare the use of ETCO_2_ ≤ 25 mmHg with qSOFA score of ≥ 2 as a predictor of mortality or diagnosis of severe sepsis in prehospital patients with suspected sepsis.

## METHODS

### Design and Setting

We conducted a retrospective cohort study among patients transported by a single emergency medical services (EMS) system to several regional hospitals during a one-year period from July 2014 through June 2015 in Orange County, Florida. The institutional review board at the participating hospitals approved the study protocol.

Inclusion criteria consisted of any case where a “sepsis alert” was activated by prehospital personnel. Per the Orange County EMS system (OCEMS) protocols, a sepsis alert is called when an adult patient (≥ 18 years) has a suspected infection, two or more of the following systemic inflammatory response syndrome (SIRS) criteria (temperature > 38° C or < 36° C, heart rate > 90 beats/min, or respiratory rate > 20 breaths/min) and an ETCO_2_ level ≤ 25 mmHg. The protocol was established immediately prior to the study period; during the roll-out time, education was provided in the form of a short, online training module. However, there were variations in protocol compliance. For example, in 42% of the sepsis alerts, ETCO_2_ values were > 25 mmHg. For the purposes of this study, the activation of the “sepsis alert” protocol defined our cohort of subjects with “suspected sepsis.” Exclusion criteria included pediatric patients (< 18 years old) and patients without available hospital records.

### Data Collection

Initial out-of-hospital data documented by first-arriving EMS personnel including SBP, respiratory rate (RR) and ETCO_2_, were obtained using LIFEPAK® 15 multi-parameter defibrillator/monitors. Prehospital measurement of ETCO_2_ is a standard practice performed by paramedics in the OCEMS via Microstream™ capnography using LIFEPAK® 15 devices (PhysioControl, Redmond, WA). ETCO_2_ was recorded when capnographic wave peaks were at a constant end-tidal for 3–5 respirations as directed by protocol. All included patients were spontaneously breathing at the time of evaluation.

We obtained patient age, gender, race, ETCO_2_, RR, SBP, and GCS from prehospital run reports. Patient mortality, admission to hospital or ICU, initial ED vital signs, pertinent past medical history, principal and admitting diagnoses defined by *International Classes of Disease, Ninth Revision, Clinical Modification* (ICD-9) codes, were obtained from the hospital chart. qSOFA scores (GCS < 15, SBP ≤ 100 mm Hg, and RR ≥ 22 breaths per minute) were calculated from the data collected from prehospital run reports. We used the ICD-9 principal diagnosis to define the diagnosis of “sepsis” or “severe sepsis.” The chart reviewers were not blinded to the primary or secondary outcomes; however, only objective, complete data were abstracted from the charts. Records were linked by manual archiving of EMS and hospital data.

The primary outcome was the relationship between ETCO_2_ and qSOFA scores and hospital mortality. The secondary outcome was diagnosis of sepsis or severe sepsis upon hospital admission.

### Analysis

We described data using means and proportions with 95% confidence intervals (CI). Data were assessed for variance and distribution and comparisons between groups were performed using Fisher’s exact test and independent sample t-tests with pooled or separate variance as appropriate. We constructed receiver operating characteristics (ROC) curves to assess the performance of ETCO_2_, and qSOFA for predicting severe sepsis and mortality. Significance was set at 0.05. We analyzed data using STATA (StataCorp, College Station, TX).

## RESULTS

Over the study period, 330 sepsis alerts were activated, 289 patients had complete prehospital and hospital records allowing for analysis for the primary outcome, and 287 patients had enough available records for analysis of the secondary outcome. Of the 203 patients with a final diagnosis of sepsis, 86 had a final diagnosis of severe sepsis, and among those 25 patients died. Patients with severe sepsis had lower ETCO_2_ values and higher serum lactate levels (see Table). There was a varied distribution of qSOFA scores; however, those with a score of 3 were more likely to be diagnosed with severe sepsis (see Table).

We constructed ROC curves to determine the accuracy of prehospital ETCO_2_ levels and qSOFA scores for predicting outcomes when a sepsis alert was activated. The area under the ROC curve predicting mortality was 0.69 for ETCO_2_ (95% CI [0.59–0.80]; p=0.001) and 0.57 for qSOFA (95% CI [0.44–0.69]; p=0.277, see Figure 1A). Combining ETCO_2_ and qSOFA scores resulted in an area under the ROC curve of 0.70 (95% CI [0.59–0.82]; p=0.001). The area under the ROC curve predicting sepsis was 0.66 for ETCO_2_ (95% CI [0.59–0.72]; p<0.001) and 0.61 for qSOFA (95% CI [0.54–0.68]; p=0.002, see Figure 1B). Combining ETCO_2_ and qSOFA scores resulted in an area under the ROC curve of 0.68 (95% CI [0.62–0.74]; p<0.001). The area under the ROC curve predicting severe sepsis was 0.78 for ETCO_2_ (95% CI [0.72–0.84]; p<0.001) and 0.69 for qSOFA (95% CI [0.62–0.75]; p<0.001, see Figure 1C). Combining ETCO_2_ and qSOFA scores resulted in an area under the ROC curve of 0.81 (95% CI [0.75–0.86]; p<0.001).

To better establish the effectiveness of the designed cut-off values for both outcome predictors, we performed comparisons between ETCO_2_ ≤ 25 mmHg and qSOFA scores of ≥ 2. Sensitivity and specificity for ETCO_2_ as a mortality predictor was higher, 80% (95% CI [59–92]) vs. 68% (95% CI [46–84]), and 42% (95% CI [36–48]) vs. 40% (95% CI [34–46]), respectively, than qSOFA score. Using both ETCO_2_ and qSOFA scores resulted in a sensitivity of 60% (95% CI [39–78]) and a specificity of 62% (95% CI [55–67]). Using either ETCO_2_ or qSOFA score increased the sensitivity of our screening tool to 88% (95% CI [68–97]); however, this resulted in a specificity of just 20% (95% CI [16–26]).

## DISCUSSION

While both ETCO_2_ values and qSOFA scores are easily obtainable within the current system, this study suggests that ETCO_2_ may have a higher discriminatory power to predict mortality and severe sepsis in potentially septic prehospital patients. Adding qSOFA scores to the ETCO_2_ protocol for identifying sepsis slightly increased sensitivity, but dramatically decreased specificity; thus, it did not add value to the existing screening tool. However, these data suggest that qSOFA may be predictive of sepsis and severe sepsis, providing an outcome predictor in austere environments or where capnography is unavailable.

Studies have shown relationships between ETCO_2_ and disease severity in patients with shock,^11^ sepsis,^7^,^8^,^12^ and trauma.^13^–^15^ ETCO_2_ is decreased due to respiratory compensation (hyperventilation) in acidotic states, and poor perfusion of alveoli in the setting of cryptic and frank shock. One advantage of ETCO_2_ relative to serum lactate is that it can be measured immediately and noninvasively, making it a simple, clinically useful outcome predictor for prehospital providers. The qSOFA score uses several traditionally measured variables to predict organ failure and shares the advantage of immediate and non-invasive calculation. Some of the overlap in predictive value between the two measures may be due to the inclusion of hyperventilation (which may lead to reduced ETCO_2_) in the calculation of the qSOFA score. The current study suggests that while qSOFA scores may assist in predicting sepsis and severe sepsis in the prehospital setting, ETCO_2_ levels had a slightly higher discriminatory power for poor outcomes.

The qSOFA score was created as part of the approach taken by the Third International Consensus Definitions for Sepsis and Septic Shock to redefine sepsis, with an emphasis on using organ failure to measure severity rather than systemic inflammation.^10^ Traditional sequential organ failure analysis (SOFA) score is calculated using variables that include laboratory analysis, and is trended over time. Interestingly, increased SOFA scores correlate with decreased ETCO_2_ levels in patients with suspected sepsis.^12^ The qSOFA score was created to provide a tool for emergency providers without access to all of the variables required for SOFA scoring. This study suggests the qSOFA score may be useful as a prehospital sepsis screening tool. The redefined definitions no longer separate the disease process into “sepsis” and “severe sepsis,” only recognizing “sepsis and “septic shock.”^10^ Since the current data were collected and analyzed prior to this refined definition, we used ICD-9 codes for “sepsis” and “severe sepsis.” While the current study suggests both ETCO_2_ and qSOFA may assist prehospital providers in identifying septic patients, further study is necessary to determine the utility of prehospital outcome predictors in relation to the new definitions.

## LIMITATIONS

There are several limitations to this study. First, the qSOFA scores were retrospectively calculated. In addition, the sepsis alert protocol used was in the initiation phases during data collection, so suspicion of sepsis may not have been as high by paramedics as it is now that more training has been provided. Of note, the most difficult and subjective portion of diagnosing sepsis - both in the field and in the hospital - remains the clinical diagnosis of suspected infection, which neither ETCO_2_ nor qSOFA alone can assist with.

## CONCLUSION

The findings of the current study suggest that ETCO_2_ performed slightly better than qSOFA scoring as a predictor of mortality from severe sepsis and the diagnosis of severe sepsis in prehospital patients with suspected sepsis. Further, prospective validation is necessary to determine the utility of qSOFA as an outcome measure applied to a wide cohort of potentially septic, prehospital patients.

## Figures and Tables

**Figure 1A:**
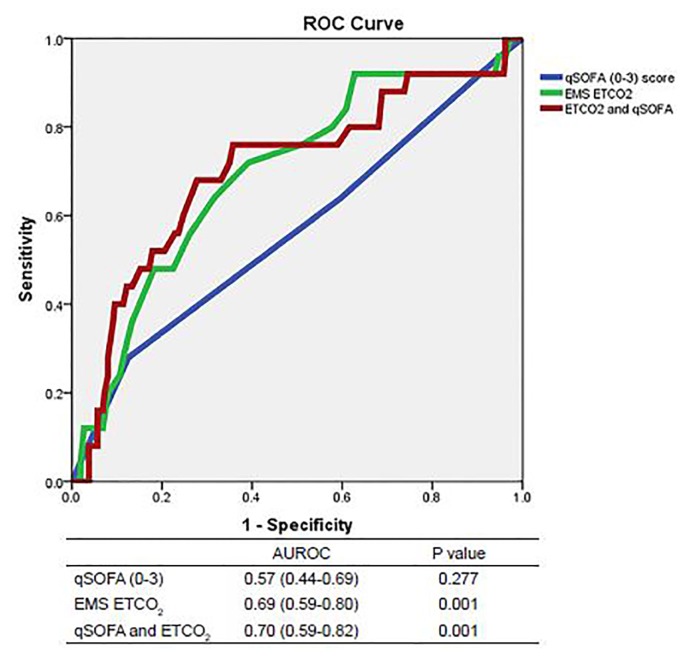
Receiver operating characteristic (ROC) curves for predicting mortality. *AUROC*,area under receiver operating characteristic curve; *qSOFA*, quick Sequential Organ Failure Assessment, *EMS*, emergency medical services; *ETCO_2_*, end-tidal carbon dioxide.

**Figure 1B:**
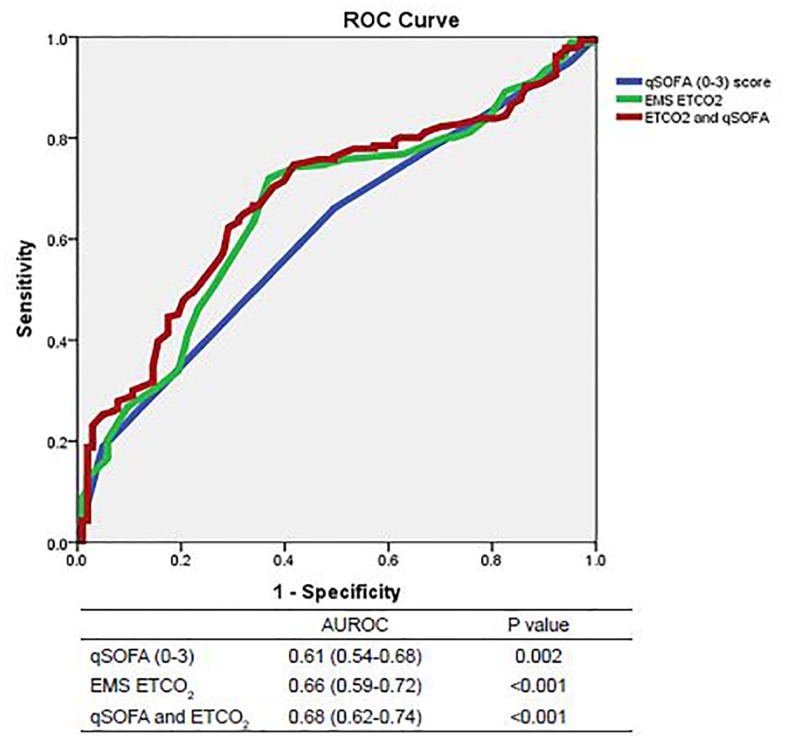
Receiver operating characteristic (ROC) curves for predicting sepsis. *AUROC*,area under receiver operating characteristic curve; *qSOFA*, quick Sequential Organ Failure Assessment, *EMS*, emergency medical services; *ETCO_2_*, end-tidal carbon dioxide.

**Figure 1C:**
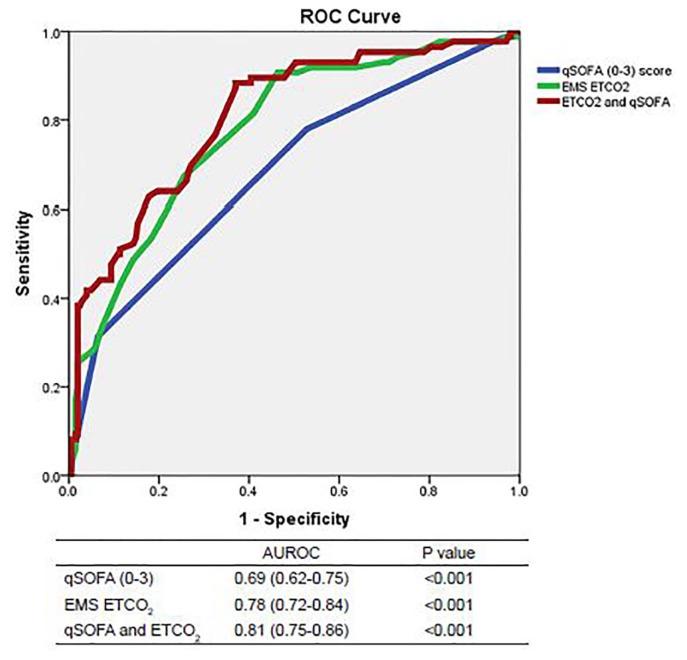
ROC curves for predicting severe sepsis. *AUROC*,area under receiver operating characteristic curve; *qSOFA*, quick Sequential Organ Failure Assessment, *EMS*, emergency medical services; *ETCO_2_*, end-tidal carbon dioxide.

**Table:** Demographics of patients with a final diagnosis of sepsis.

	Sepsis N=203	Severe sepsis N=86	Total N=289	P value
Age (n=289)	69 (SD18)	74 (SD15)	70 (SD17)	0.034
Gender (female) (n=289)	108 (53%)	41 (48%)	149 (52%)	0.440
Admitted (n=287)	193 (96%)	85 (100%)	278 (97%)	0.062
Admitted to ICU (n=285)	49 (25%)	50 (59%)	99 (35%)	<0.001
Hospital mortality (n=288)	9 (5%)	16 (19%)	25 (9%)	<0.001
Admitting diagnosis (n=287)				
Abdominal/GI	14 (7%)	2 (2%)	16 (6%)	
Altered mental status	19 (10%)	6 (7%)	25 (9%)	
Cardiac/vascular	3 (2%)	1 (1%)	4 (1%)	
Respiratory	35 (17%)	8 (9%)	43 (15%)	0.009
Infection	85 (42%)	60 (70%)	145 (52%)	
Neurologic	3 (2%)	0 (0)	3 (1%)	
Metabolic/endocrine	9 (5%)	2 (2%)	11 (4%)	
Renal/urinary	26 (13%)	4 (5%)	30 (11%)	
Other	7 (4%)	3 (4%)	10 (4%)	
At least 2 SIRS criteria	187 (93%)	84 (98%)	271 (94%)	0.108
qSOFA score				
0	12 (6%)	2 (2%)	14 (5%)	
1	84 (41%)	17 (20%)	101 (35%)	<0.001
2	94 (46%)	40 (47%)	134 (46%)	
3	13 (6%)	27 (31%)	40 (14%)	
ETCO_2_ [95% CI]	28 [27–29]	19 [18–22]]	25 [24–16]	<0.001
Lactate (n=228)	1.9 [1.8–2.1]	5.4 [4.8–6.2]	3.2 [2.8–3.5]	<0.001
HCO_3_ (n=259)	24 [23–24]	20 [19–22]	23 [22–23]	<0.001

*ICU*, intensive care unit; *GI*, gastrointestinal; *SIRS*, systematic inflammatory response syndrome; *qSOFA*, quick Sequential Organ Failure Assessment.
